# The human μ-opioid receptor gene polymorphism (A118G) is associated with head pain severity in a clinical cohort of female migraine with aura patients

**DOI:** 10.1007/s10194-012-0468-z

**Published:** 2012-06-30

**Authors:** S. Menon, R. A. Lea, B. Roy, M. Hanna, S. Wee, L. M. Haupt, L. R. Griffiths

**Affiliations:** Genomics Research Centre Griffith Health Institute, Griffith University Gold Coast, Parklands Drive, Southport, QLD 4222 Australia

**Keywords:** Migraine, Opioid, *OPRM1* gene, A118G SNP

## Abstract

Migraine is a painful and debilitating, neurovascular disease. Current migraine head pain treatments work with differing efficacies in migraineurs. The opioid system plays an important role in diverse biological functions including analgesia, drug response and pain reduction. The A118G single nucleotide polymorphism (SNP) in exon 1 of the μ-opioid receptor gene (*OPRM1*) has been associated with elevated pain responses and decreased pain threshold in a variety of populations. The aim of the current preliminary study was to test whether genotypes of the *OPRM1* A118G SNP are associated with head pain severity in a clinical cohort of female migraineurs. This was a preliminary study to determine whether genotypes of the *OPRM1* A118G SNP are associated with head pain severity in a clinical cohort of female migraineurs. A total of 153 chronic migraine with aura sufferers were assessed for migraine head pain using the Migraine Disability Assessment Score instrument and classified into high and low pain severity groups. DNA was extracted and genotypes obtained for the A118G SNP. Logistic regression analysis adjusting for age effects showed the A118G SNP of the *OPRM1* gene to be significantly associated with migraine pain severity in the test population (*P* = 0.0037). In particular, G118 allele carriers were more likely to be high pain sufferers compared to homozygous carriers of the A118 allele (OR = 3.125, 95 % CI = 1.41, 6.93, *P* = 0.0037). These findings suggest that A118G genotypes of the *OPRM1* gene may influence migraine-associated head pain in females. Further investigations are required to fully understand the effect of this gene variant on migraine head pain including studies in males and in different migraine subtypes, as well as in response to head pain medication.

## Introduction

Migraine is a chronic neurovascular disorder that is characterised by unilateral throbbing headache associated with features such as sensitivity to light and sound, nausea, vomiting and neurological disturbances [1, 2]. The condition, which is classified into 2 main subtypes, migraine with aura (MA) or without aura (MO), has a major impact on the quality of life of patients and their families [3–5]. The pathogenesis of pain in migraine is still not fully understood, but three key factors considered to play a part include disruption of: the cranial blood vessels, the trigeminal innervation of the vessels, and the reflex connection of the trigeminal system in the cranial parasympathetic outflow [1]. The main aim of acute migraine treatment, such as non-steroidal anti-inflammatory drugs (NSAIDs), aspirin and triptans, are rapid pain relief, improvement of associated symptoms and prevention of migraine recurrence within 24 h [6]. Not all migraineurs find the current medication options effective, which perhaps highlight the multifactorial pathophysiology of migraine and suggest that individual environmental and/or genetic factors may influence treatment response [4].

An individual’s pain experience is shaped by psychological, behavioural and genetic factors [7]. Although there is no conclusive evidence for the heritability of pain sensitivity in humans, studies have shown evidence that a variety of thermal, mechanical and chemical pain-producing stimuli are genetically influenced in humans [8, 9]. Twin studies comparing the concordance rates of pain-related traits in monozygotic versus dizygotic twins, in order to separate the genetic and environmental factors, have estimated heritability as 39–58 % for migraine [10–12], 55 % for menstrual pain [13], 50 % for back pain [14] and 21 % for sciatica [15]. In addition to twin studies, recent research has examined the association between single nucleotide polymorphisms (SNPs) of specific genes and experimental pain responses. Kim et al. [16] in their research reported heritability estimates of 22–46 % across three pain modalities and found an association of a δ-opioid receptor gene (*OPRD1*) SNP with thermal pain response among men, but not women. Zubieta et al. [17] reported a SNP in the catechol-*O*-methyltransferase gene (*COMT*) to be associated with pain self-report and pain-induced brain μ-opioid receptor binding during experimental muscle pain induction. Thus, though limited, there is some evidence for a genetic contribution to human pain response.

The μ-opioid receptor gene (*OPRM1*) is located on the human chromosome 6q24-25. The OPRM1 receptor is the site of action for many endogenous opioid peptides and a significant target for opioid analgesics [18, 19]. Opioid analgesics, such as morphine and codeine have been used by clinicians to treat severe cases of migraine headache [20]. The *OPRM1* gene has been widely studied and reported to be associated with pain sensitivity in humans [21–23]. A commonly studied *OPRM1* gene SNP is the A118G in exon 1 that causes an Asn40Asp substitution at a putative glycosylation site in the extracellular domain [24]. The A118G SNP in the *OPRM1* gene has been widely investigated and associated with elevated pain responses and decreased pain thresholds [21, 22]. Some functional consequences of this SNP include lower mRNA and protein expression levels with the G118 variant [25]. The G118 variant also increases receptor affinity of β-endorphin by threefold [24], which could potentially be the mechanism by which this SNP may affect pain sensitivity [26].

Studies that investigated the relationship between pain thresholds and analgesic responses to opioid administration for the A118G SNP have in most instances associated the G allele with decreased pain threshold and reduced response to opioids for patients receiving post-operative care or chronic pain [22, 27–29]. The *OPRM1* gene ranks highly on all three criteria introduced for prioritizing candidate genes in association studies investigating pain, namely: strength of evidence supporting involvement in pain sensitivity, frequency of the specific variant, and evidence for functional consequence of the SNP [26, 30]. However to date, no studies have reported an association between the A118G SNP and head pain associated with migraine, ranked 19th in the world’s most disabling illness by the World Health Organisation [4].

Studies have shown that certain gene mutations influence treatment suitability and options for some migraine subtypes [31–33]. Understanding the effect of the A118G SNP on pain sensitivity could aid in optimising pain treatment for migraineurs. This study focussed on the A118G SNP and migraine-associated head pain severity. A total of 153 females with MA were studied to investigate the association between A118G genotypes and head pain severity. We selected only females because the disease is far more prevalent in females and a far greater number of female patients present at clinics with chronic head pain associated with migraine [34]. Fillingm et al. (2000) reviewed literature in sex, gender and pain and found substantial sex differences in clinical and experimental procedure [34]. A recent review also highlighted the consistent literature showing higher prevalence of headaches and migraine among women, who also reported higher clinical pain intensities than men for a number of disease entities [35]. There was a focus on MA so as not to introduce confounding due to genetic and phenotypic differences between the migraine subtypes.

## Methods

### Participants

This was a retrospective clinical epidemiologic study focussing on a sample of 153 adult females diagnosed with MA. All participants were Caucasian residents of Australia (East Coast) with European ancestors from various locations within the British Isles and other parts of Europe. Participants completed a detailed questionnaire that was administered by a qualified diagnostician through Griffith University’s Genomics Research Centre (GRC) clinical trials unit. Participants were included if they had suffered migraine for over 20 years and had a current diagnosis of MA (>90 % of their migraine attacks were associated with aura), and a 1-year history of severe, long lasting attacks (at least 4 attacks lasting more than 48 h) and had a family history of migraine.

A whole blood sample was collected from each participant, for genomic DNA extraction from white blood cells, according to an approved protocol for experimentation on human participants. All participants gave their informed and written consent for genetic analysis of their blood samples. Participant’s medication use; frequency, quantity and treatment compliance were also recorded. Participants were excluded if they had been diagnosed with a clinically recognized cardiovascular or neuropsychiatric condition such as depression or schizophrenia. The study was approved by the Griffith University Ethics Committee for Experimentation on Human Participants.

### Assessment of head pain associated with migraine

All participants were assessed for migraine-related head pain using the Migraine Disability Assessment Score (MIDAS) instrument, which provides a measure of pain severity, headache frequency and disability [31, 36, 37]. Studies have shown that this is a valid and clinically useful instrument for assessing health-related quality of life in migraineurs [36, 37].

Based on the 5-question MIDAS rating, participants were arbitrarily categorized into a ‘low’ disability group if they had a MIDAS rating of 0–10 and into a ‘high’ disability group if they had a MIDAS rating greater than 11 [31, 36, 37]. Question 6 of the MIDAS instrument is on migraine frequency. This is measured as number of days with headache over a 3-month period [31, 36, 37]. The outcome variable assessed in this study was migraine head pain severity, question 7 on the MIDAS instrument, which is measured as a self-reported pain score (based on a scale of 0–10). The MIDAS migraine head pain severity question is as stated “B. On a scale of 0–10, on average how painful were these headaches? (Where 0 = no pain at all, and 10 = pain as bad as possible)” [31, 36, 37]. Patients were asked to provide a head pain assessment prior to taking any medication or treatment for their migraine attacks. Scoring was retrospectively based on the patient’s untreated migraine attacks in the 3-month period prior to recording.

### OPRM1 A118G genotyping

**Fig. 1:**
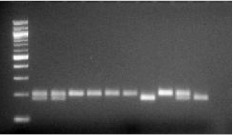
Agarose gel picture illustrating the different A118G genotypes identified by RFLP using the *Bst UI* restriction enzyme. *Column 1* is a 100 bp marker, *column 2* shows a sample homozygote for the mutant G118 allele, *column 3* shows a sample heterozygote for the A118G SNP and *columns* 4–8 are samples heterozygous for the wild-type A118 allele

Genomic DNA was obtained from leucocytes following a salting out method as previously described by Miller et al. [38]. Genotyping of the A118G SNP was conducted by polymerase chain reaction (PCR). The A118G SNP was amplified using the forward primer (5′-GGTCAACTTGTCCCACTTAGAT *C* GC- 3′) which was designed to create a restriction site for the enzyme *Bst* UI when the G118 allele is present and reverse primer (5′-AATCACATACATGACCAGGAAGTTT-3′) [39]. The PCR conditions used were an initial denaturation at 94 °C for 3 min, followed by 38 cycles of 94 °C for 30 s, 60 °C for 1 min, 72 °C for 1 min, with a final extension of 72 °C for 10 min [19]. Genotyping for the A118G polymorphism was performed by digesting PCR products with the *Bst UI* restriction enzyme (New England Biolabs, Beverly, MA, USA). The enzyme recognises the CGCG sequence and reacts at 60 °C. The digested PCR products were resolved using a 3 % agarose gel, stained with ethidium bromide and visualised under ultra-violet light. The *Bst UI* digestion of the A allele gave a band of 193 bp. The G allele gave 164 and 24 bp fragments. The results of each genotype were confirmed in randomly selected subset of individuals (10 %) via sequencing using an ABI-3130 Genetic Analyser. Sequence electrophoretograms were examined visually using the ABI Sequencing Analysis software 5.0 to determine the alleles of the *OPRM1* A118G SNP (Fig. 1).

**Fig. 2:**
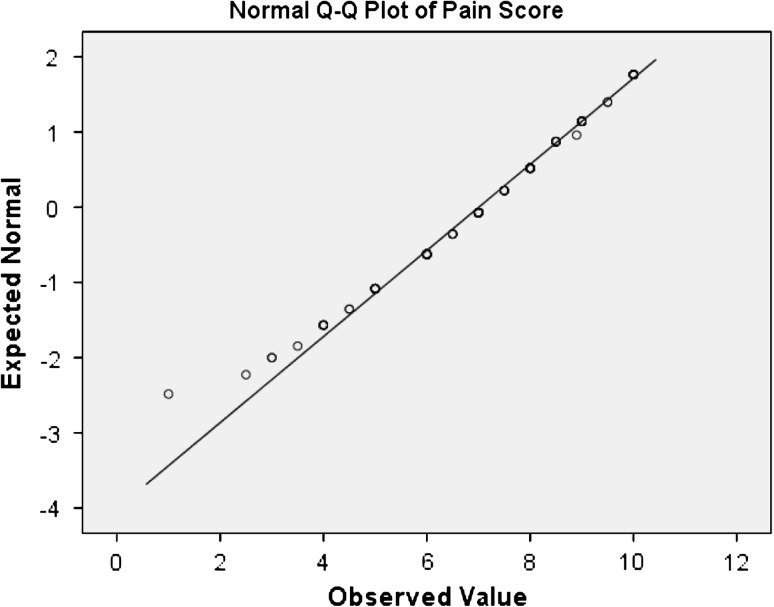
Normality plot of pain scores for the 153 female MA sufferers in the study

### Statistical analysis

Summary statistics was calculated for migraine head pain severity in each of the three genotypic groups (A/A, A/G, G/G). Hardy–Weinberg equilibrium (HWE) was verified for observed genotype frequencies of the *OPRM1* A118G SNP to detect deviation from the expected genotype distribution and to detect genotyping error. Data for migraine head pain severity in the AG and GG genotypes were added together and presented as one category to test the hypothesis that G allele carriers have a higher average migraine head pain severity compared to non-carriers as suggested by previous studies [21, 22]. Pain score data was first evaluated for normality of the distribution and subsequently dichotomised into two categories based on the 50th percentile of the distribution. All patients were assigned a pain score code of 0 or 1, whereby 0 = low pain (a score <7) and 1 = high pain (a score ≥7). To test the effects of genotype on migraine pain severity, the χ^2^ test was performed using the pain score categories described above. Odds ratios (OR) with their associated 95 % confidence intervals (95 % CI) were calculated using logistic regression analysis with age included as a covariate. The threshold of statistical significance was defined as 0.05, and all analyses were performed using the Statistical Package for Social Sciences (SPSS version 17.0).

## Results

Among the 153 MA patients that were analysed in this study, there were 119 (77.3 %) wild-type homozygotes (A/A), 32 (26.9 %) heterozygotes (A/G) and 2 (1.7 %) mutant homozygotes (G/G). The genotype distribution for the group did not deviate from HWE (*P* > 0.9). Based on previous research and the hypothesis that the G allele specifically is associated with increased pain [21, 22], the A/G and the G/G genotypes were grouped together and compared against the A/A genotypes. This analysis showed that 64.7 % of the G carriers had high head pain severity scores compared to 37 % of the A/A carriers. Normality plots indicated that the pain score data was not normally distributed (*P* < 0.05) and substantially asymmetrical (Fig. 2). For this reason the pain scores were dichotomised as described in “Methods” before further analysis. Logistic regression analysis showed that the G allele (i.e. A/G and G/G) was significantly associated with ‘high’ migraine head pain severity (OR = 3.125, 95 % CI = 1.41, 6.93, *P* = 0.0037) (Table 1). Age was included as a covariate because of possible association of age on head pain score (Table 2).

**Table 1:** Clinical characteristics of patient group based on their OPRM-1 A118G genotype

Variable	A/A carriers	A/G, G/G carriers
No. of patients	119	34
Age in years, mean (±SD)	43 (±14)	44 (±14)
Head pain score, median (range)	7 (1–10)	8 (3–10)
High head pain (%)	37	64.7

**Table 2:** Head pain scores for female MA patients by *OPRM1* gene, A118G SNP genotype

Group	Genotypes			
Migraine pain severity score	AA	AG/GG	Odds ratio*	*P* value*
Low score (*n* %)	75 (63)	12 (35.2)	3.125	0.0037
High score (*n* %)	44 (37)	22 (64.7)		
Total	119	34		

Chi-squared (χ^2^) analysis revealed significant association between genotypic frequencies for the A118G SNP in the *OPRM1* gene and high scores for head pain in MA females. Values are total number genotypes. Significance was taken at *P* ≤ 0.05

* Odds ratio and *P* value adjusted for age

## Discussion

This study investigated if the *OPRM1* gene, A118G SNP was associated with migraine head pain severity in females. Specifically, the aim of this study was to elucidate the propensity of the G allele in affecting head pain perception in MA sufferers. A total of 153 female MA patients were genotyped for the *OPRM1* A118G SNP. The major finding of this study was that genotypes of the A118G SNP were associated with head pain severity in female MA sufferers, whereby G118 allele carriers had demonstrably higher likelihood of being high pain sufferers compared to the homozygous A118 allele carriers.

The multi-factorial pathophysiology of migraine has complicated diagnosis and treatment. A number of genes, causative mutations and susceptibility variants have been reported to work separately or in synergy to result in certain types of migraine [40]. Further studies on the genetic contribution to migraine are necessary to understand the complex pathophysiology of this disease. The human *OPRM1* gene is one of the primary candidates for pharmacogenetic study and is the site of target for opioid analgesics [20]. The relationships between altered pain thresholds and responses to analgesic opioid administration and the A118G SNP in the *OPRM1* gene have been well investigated and characterised [29]. The G118 allele has been associated with increased pain responses and reduced response to opioid analgesics in patients receiving treatments for post-operative or chronic pain, in a variety of populations over the last couple of years [26–28, 41–43].

A recent study that investigated the association of A118G with self-rated postoperative pain and the amount of self-administered morphine in females reported that the A118G variant was associated with higher pain scores [22]. Contrastingly, Fillingim et al. [26] reported that healthy volunteers with the G118 allele receiving different experimental pain procedures had reduced pain responses to pressure. Additionally the males in the experimental group with the G allele rated thermal pain lower than those with the A allele. However, the females carrying the G allele in the experimental group reported elevated pain response following thermal pain administration, consistent with previous studies [26]. The change of quantity and binding affinity of the μ-opioid receptors brought about by the A118G SNP has been implicated for the observed effects of this SNP [19]. Further studies, however, are still warranted to understand the exact molecular effect of the A118G SNP on the *OPRM1* gene function that may elucidate the observed clinical effects of this SNP.

This is the first study to investigate the association between the *OPRM1* A118G SNP and migraine head pain severity. The limitations of this study deserve mention. A notable number of studies support the contention that the human *OPRM1* is a pain-relevant gene and that the A118G SNP in the *OPRM1* gene is potentially related to altered opioid requirements for achieving analgesia and/or altered pain perception mechanism. Hence the current study focused only on the A118G SNP in the *OPRM1* gene as a prime candidate in investigating migraine pain. However, the A118G SNP is only one of the more than 100 known SNPs in the *OPRM1* gene and therefore further studies are required to investigate other candidate, albeit rare SNPs to understand their influence on pain associated with migraine. Secondly because, this is the first study associating the *OPRM1* gene with migraine pain severity, replication in additional, larger populations is necessary to enhance confidence in results. The fact that allele frequency estimates of the A118G SNP in current study population were similar to that of studies reported previously suggests that the population is homogeneous in ancestry and the potential for confounding is minimal, it is still not exempt from stratification completely.

Most of the participants in this study took some form of pain relieving medication which are essentially taken during a migraine attack and are designed to abort symptoms that have already begun. The common classes of medicines taken by the participants of this study include ergotamine, triptan and simple analgesics (non-steroidal anti-inflammatory drugs, acetylsalicylic acid, paracetamol). Pain scoring was retrospectively based on the patient’s untreated migraine attacks in the 3-month period prior to recording and therefore it was accepted that the pain score recorded was not influenced by medication effects. However, that is not to say that medication taken during a migraine attack could not have lessened the associated symptoms (i.e. head pain) before it reached a threshold that required the participant to give a higher pain score.

Lastly, this study has been confined to only female MA sufferers and as yet we do not know if this finding will be replicated in female MO migraineurs and in male migraineurs. Therefore, caution should be exercised when interpreting the results of this study. As such, this study has taken the first step towards investigating the association between the A118G SNP in the *OPRM1* gene and migraine pain severity and has provided initial evidence that the presence of the G118 allele may increase the severity of migraine pain.

In conclusion the results from this study suggest that the A118G genotypes of the OPRM1 gene may influence migraine-associated head pain in females. Further investigations are needed to fully understand the effect of this gene variant on migraine head pain including studies in different migraine subtypes and in males, as well as in response to head pain medication to optimise treatment options.
